# Using antibuoyant capacities to account for species importance when modelling biodiversity

**DOI:** 10.1098/rsfs.2025.0062

**Published:** 2026-08-28

**Authors:** Gleb Beliakov, Simon James, Dale G. Nimmo, Jian-Zhang Wu

**Affiliations:** ^1^ Deakin University, Melbourne, Victoria, Australia; ^2^ Charles Sturt University, Albury, New South Wales, Australia; ^3^ Guangzhou Nanfang College, Guangzhou, Guangdong, People's Republic of China

**Keywords:** capacity, Choquet integral, antibuoyant fuzzy measure, species diversity

## Abstract

Capacities satisfying the antibuoyancy condition can be used to define Choquet integrals that adhere to the Pigou–Dalton or progressive transfers principle. As well as being a key concept in economics, where the principle underlies measures of welfare and wealth inequality, it has also often been adopted in ecology for defining indices related to species diversity. The point of difference for the Choquet integral in this setting is that it can also incorporate the differing importances of inputs and groups of inputs—not just whether they are high or low relative to the others. It makes it possible to define measures of species diversity that can place increased value on abundance of rare or keystone species, while still adhering to the Pigou–Dalton principle. In this contribution, we outline some of the key considerations for applying and constructing antibuoyant capacities in this context. Illustrative examples are provided along with an application to optimizing measures of species diversity with respect to land-use constraints. We also briefly address the behaviour of the Sugeno integral with respect to antibuoyant capacities.

This article is part of the theme issue ‘Data driven modelling for living systems’.

## 1. Introduction

Definitions for species biodiversity in ecology usually incorporate three concepts: *abundance*, which measures how many individuals there are in the population; *richness*, which is the number of different species; and *evenness*, which is a measure of how evenly spread abundance is between the different species. A common position is that diversity should be the product of evenness and richness [1]. Although the concept of richness as a count of the number of species present may be simple enough, numerous indices have been proposed for evenness, with several desirable properties investigated in, for example, [1] and [2]. The concept of evenness usually aligns with that of economic equality, with a defining feature of classic evenness indices (e.g. [3,4]) being satisfaction of the Pigou–Dalton progressive transfers principle proposed in [5], which states that the level of equality should increase with transfers of wealth from richer to poorer individuals. Shannon's entropy is also a related concept [6]. A community with no diversity would be characterized by a single abundant species, which can be seen to align with a single individual owning all the wealth in the economic context. At the other end of the spectrum, the highest level of species diversity would be achieved in communities characterized by all species being equal in abundance, corresponding with economies where all individuals are equally wealthy.

Beyond scientific interest, the quantification of species diversity and related concepts is important for policymaking, allowing communities to be compared in terms that may reflect conservation value, or to predict the impact that land management decisions may have. In [7,8], optimization of biodiversity indices based on aggregation functions was used to evaluate the land-sparing and land-sharing approaches to population density planning. These models assumed species to be equally important; however, community and expert perceptions may favour conservation efforts towards some species more if they are rare or play an important role in a particular community, for example, as a keystone species.

In the species diversity setting, the Pigou–Dalton principle [5] requires that proportional transfers from a more abundant to less abundant species (without altering the relative order of abundance) should not result in a lower evaluation of biodiversity. This is usually framed in equivalent terms for properties of evenness, for example, the second property as given in [1].

The concept of buoyancy (and its dual property, antibuoyancy) was proposed for fuzzy measures and capacities in [9,10]. This extends the property established for the weighting vectors of the symmetric ordered weighted averaging (OWA) operators in [11] to the non-symmetric case. Choquet integrals defined with respect to antibuoyant capacities satisfy the Pigou–Dalton principle and also lead to simpler optimization problems.

Here, we showcase the suitability of antibuoyant capacities in the modelling of biodiversity, contending that this property facilitates measures of species diversity that are consistent with the Pigou–Dalton principle, but that such capacities also allow experts to factor in varying importances of different species. To our knowledge, there are no existing models that incorporate both of these properties simultaneously. We will provide an overview of the antibuoyancy property for capacities and how this property influences the behaviour of the Choquet and Sugeno integrals. Although the Sugeno integral has some useful properties in this context, ultimately it fails in adherence to the Pigou–Dalton principle, even when the underlying capacity is antibuoyant. We therefore focus on applications involving the Choquet integral, looking at the construction of antibuoyant capacities and solutions to the resulting optimization problems that seek to maximize species diversity subject to other resource constraints.

The structure of the paper will be as follows: in §2, we will give an overview of capacities and the Choquet and Sugeno integrals, before outlining the definition and properties of antibuoyant capacities in §3. In §4, we provide illustrative examples demonstrating how the Choquet integral with respect to antibuoyant capacities can be applied in modelling species diversity. We then show how such measures can be constructed in §5 before looking at some optimization-based applications in §6. We conclude with some final remarks in §7.

## 2. Capacities, the Choquet and Sugeno integrals

Here, we will provide the mathematical definitions for capacities as well as the Choquet and Sugeno integrals, which use capacities in their calculation. Some finer details of the theory are deferred to appendix A, or can be found in cited papers.

We assume we have a set N={1,2,…,n}, which may be criteria in decision-making or an indexing of species, individuals in a community, land types and so on.

**Definition 1. [12]**

*A Choquet capacity is a grounded set function $\mu :2^{N}\to R_{+}$ satisfying monotonicity with respect to set inclusion, that is, μ(A)≤μ(B) whenever A⊂B and subject to the boundary condition μ(∅)=0*.

The motivation for using set functions in aggregating inputs is to account not only for individual input importance but also for relative importances of subsets of inputs, thus factoring in possible input interactions. So the values μ(A) represent relative contribution to the total by each subset of inputs A.

We can represent capacities using a Hasse diagram as shown in figure 1. This allows us to verify the monotonicity relationship between capacity values by looking at adjacent nodes.

**Figure 1.**
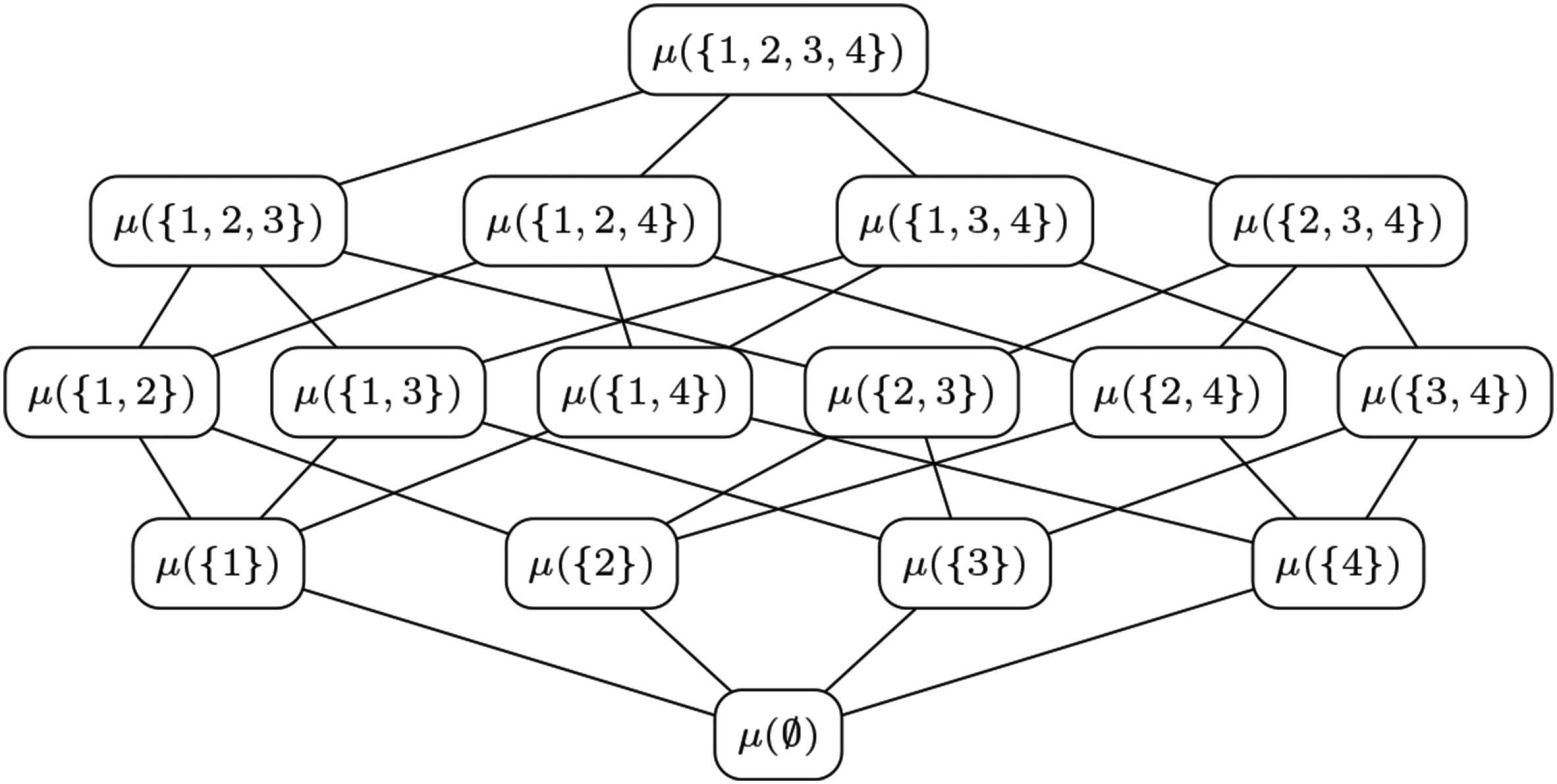
Hasse diagram with subsets indicated by edges going upward. This allows us to represent examples in a more compact format.

The Choquet integral can then be calculated as follows.

**Definition 2. [12]**

*For a given capacity μ, the discrete Choquet integral of a set of inputs $x=(x_{1},x_{2},\ldots ,x_{n})$ is given by*

(2.1) $$C_{\mu }(x)=\sum_{i=1}^{n}x_{\left(i\right)}(\mu (H_{i})-\mu (H_{i+1})),$$

*where $x_{\left(1\right)}\leq \cdots \leq x_{\left(n\right)}$ denotes an increasing permutation of the inputs, and $H_{i}=\{(i),(i+1),\ldots ,(n)\}$ is the set of corresponding elements from (i) up to (n) with $H_{n+1}=\emptyset$*.

Examples of how this calculation works are represented visually in figures 2 and 3. Suppose we have a capacity as depicted in figure 2a. The node correspondence with subsets is the same as for figure 1, that is, with the top node of 1 giving the value assigned to the subset {1,2,3,4} and the first value in the second row corresponding with {1,2,3} and so on. If the relative order of the inputs is given by $x_{1}<x_{2}<x_{3}<x_{4}$, then the inputs will be calculated using the values shown along the path indicated in bold. For $x_{1}$, the smallest input, we take the product of $x_{1}$ and $\mu (H_{1})-\mu (H_{2})=1-0.6$. This means the effective weight applied to $x_{1}$ is 0.4, then the weight applied to $x_{2}$ is 0.6−0.3=0.3 and so on. The effective weights are represented by the widths $w_{1},w_{2}$ and so on in figure 2b.

**Figure 2.**
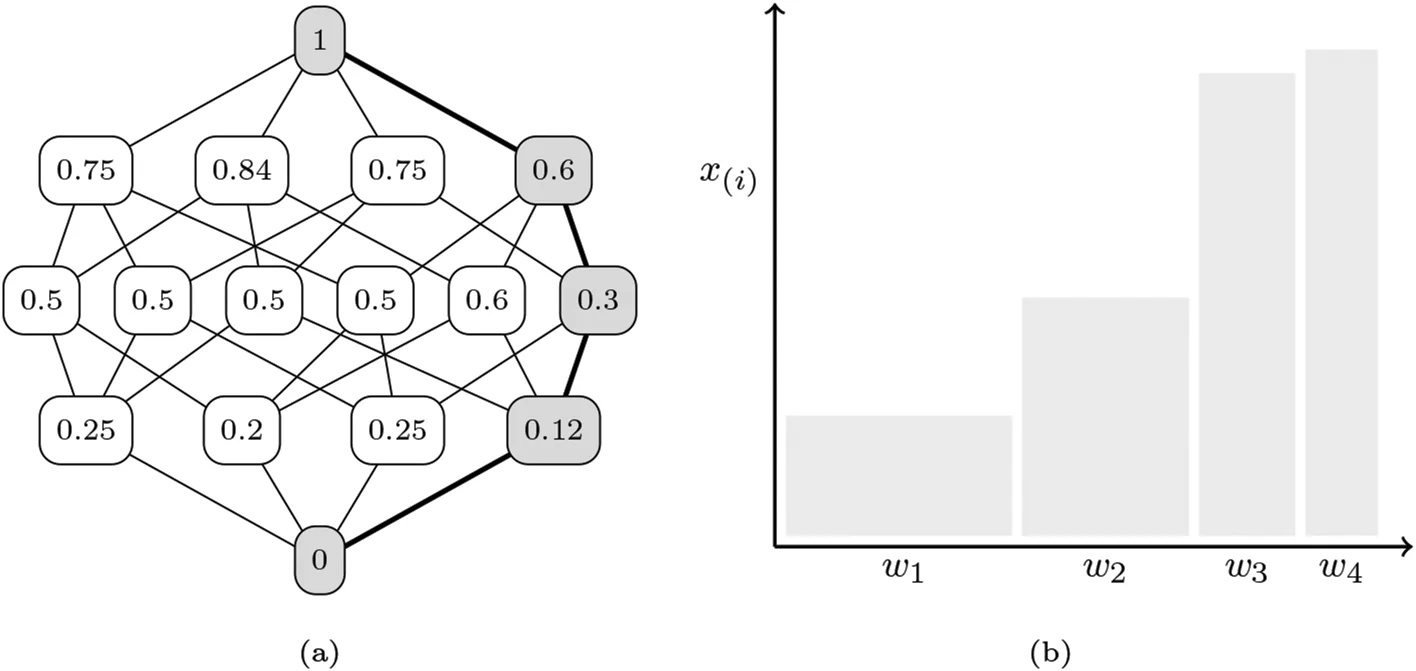
(a) Hasse diagram for an example capacity on N={1,2,3,4} with the path of calculation when $x_{1}<x_{2}<x_{3}<x_{4}$ indicated by the bold line and shaded cells. (b) A visual representation for an example set of inputs with this relative ordering, with effective weights applied to the inputs indicated by the widths of the input bars. The overall output would correspond with the sum of the products $w_{i}x_{\left(i\right)}$ and hence be given by the total area of the shaded regions.

**Figure 3.**
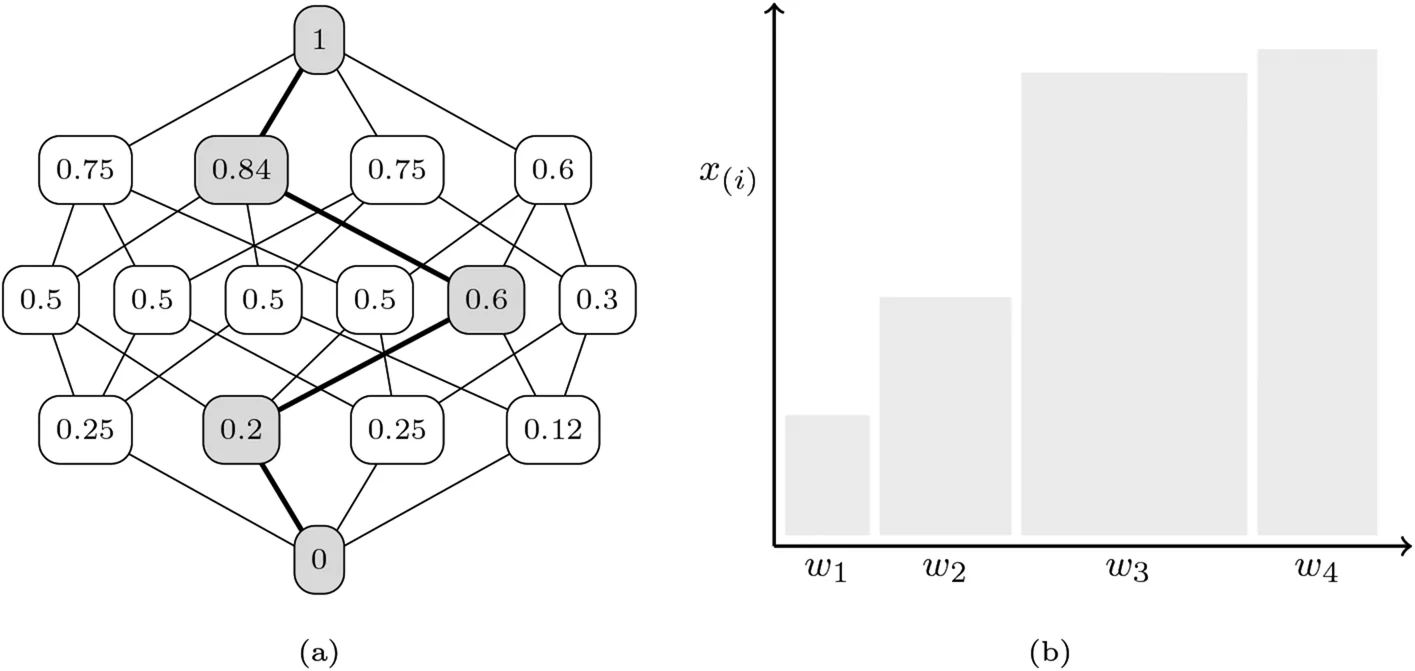
(a) The same capacity as given in figure 2, this time with the path of calculation shown for $x_{3}<x_{1}<x_{4}<x_{2}$. (b) The resulting weights applied are indicated as widths.

On the other hand, if we have $x_{3}<x_{1}<x_{4}<x_{2}$, then the weight applied to $x_{3}$ (which is now $x_{\left(1\right)}$ in this calculation) will be 1−0.84=0.16. The maximal chain used in the calculation is shown in figure 3a with the resulting weights indicated as widths in figure 3b.

The Sugeno integral [13] is another well-known aggregation function defined with respect to capacities; however, the calculation is based on minimum and maximum operations, allowing it to be applied to ordinal inputs as well as numerical inputs.

**Definition 3. [13]**

*For a capacity μ, the Sugeno integral of x is given by*

(2.2) $$S_{\mu }(x)=\max_{i\in \{1,2,\ldots ,n\}}\min(x_{\left(i\right)},\mu (H_{i})),$$

*where $x_{\left(1\right)}\leq \cdots \leq x_{\left(n\right)}$ denotes an increasing permutation of the inputs, and $H_{i}=\{(i),(i+1),\ldots ,(n)\}$ is the set of corresponding elements from (i) up to (n) as was the case for the Choquet integral*.

We can demonstrate examples of calculation using the same capacity and relative orderings as we have shown for the Choquet integral (figures 2 and 3). This time, we calculate the minimum of each pair $\left(x_{\left(i\right)},\mu (H_{i})\right)$, with the output being the maximum of these. In figure 4a, we can see that the ordering $x_{1}<x_{2}<x_{3}<x_{4}$ results in the largest minimum being obtained for the pair $(x_{\left(2\right)},\mu (H_{2}))=x_{2}$, while if the ordering is $x_{3}<x_{1}<x_{4}<x_{2}$, we have the minimum of $\left(x_{\left(3\right)},\mu (H_{3})\right)$ giving the largest value, which is equal to $\mu (H_{3})$.

**Figure 4.**
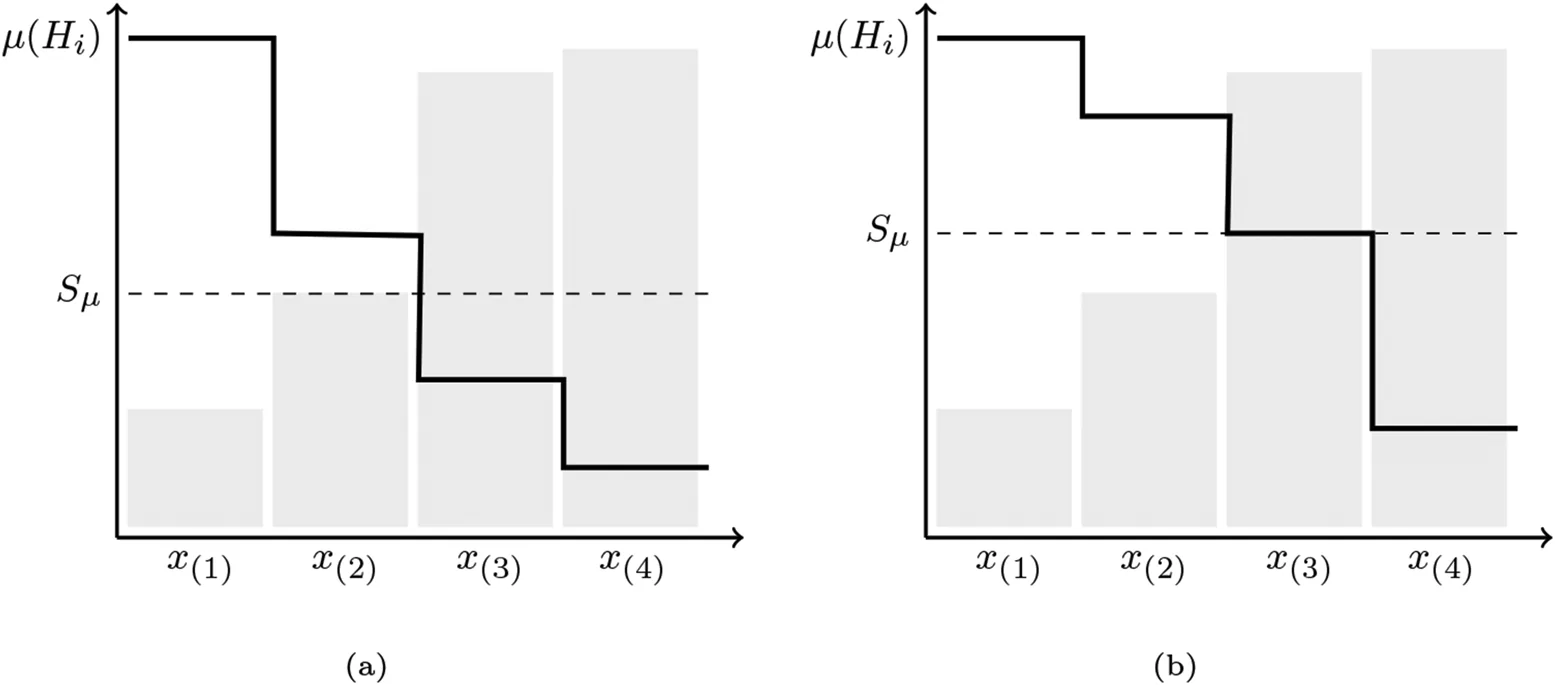
Visual representation of the Sugeno integral calculation for a set of inputs with respect to the capacity shown in figures 2a and 3a. This time the inputs are labelled as $x_{\left(1\right)},x_{\left(2\right)}$ and so on, and the $\mu (H_{i})$ values are indicated by the decreasing sequence indicated by the thick line. (a) corresponds with $x_{1}<x_{2}<x_{3}<x_{4}$ as shown in figure 2 and (b) shows the simplex $x_{3}<x_{1}<x_{4}<x_{2}$ from figure 3. The output is the dotted line, corresponding with the maximum over the minimum of each $\left(x_{\left(i\right)},\mu (H_{i})\right)$ pair.

The two mentioned fuzzy integrals combine the input values $x_{i}$ by accounting for their individual weights (the values μ({i}) at singletons), and also their interactions in all possible groups of inputs. Depending on the type of capacity used, one can ensure certain properties of fuzzy integrals, such as convexity (called submodularity), symmetry, additivity or the maximum set cardinality for incorporating inputs’ interactions. Here, we specifically focus on the Pigou–Dalton property of progressive transfers, which favours more equitable species distribution.

## 3. Antibuoyant capacities

The concept of buoyancy, and its dual property, antibuoyancy, were introduced for OWA operators in [14,15]. OWA operators are equivalent to Choquet integrals whose capacities are symmetric, that is, the capacity values are the same for all sets of the same size.

Observe that in figure 2 the weights $w_{i}$ visualized in (b) form a decreasing sequence, whereas for figure 3 they do not. An antibuoyant capacity is one which always results in sequences that are decreasing (or more formally, non-increasing), whereas a buoyant capacity is one that results in increasing sequences. In the case of antibuoyant capacities, the largest effective weight is applied to the smallest input.

For functions like the Choquet integral, the Pigou–Dalton principal requires that for a set of ordered inputs $x_{\left(1\right)}\leq x_{\left(2\right)}\leq \cdots \leq x_{\left(n\right)}$, if value is ‘transferred’ from a higher to lower input, that is, for $x_{i}<x_{j}$, we replace the respective inputs with $x_{i}+h$ and $x_{j}-h$ (as long as $x_{i}+h$ is still smaller than $x_{j}-h$), then the output should reflect a higher level of equality.

The buoyancy/antibuoyancy properties are similar to a kind of convexity/concavity on the function. In the case of symmetric capacities, antibuoyancy and convexity are equivalent [16]; however, convexity is a weaker property for more general capacities and does not ensure that the Pigou–Dalton property is satisfied. It was shown in [9] that antibuoyancy is the required property.

We provide the following definition of antibuoyancy in terms of the effective weights obtained from each $\mu (H_{i})-\mu (H_{i+1})$ in the Choquet integral.

**Definition 4. [9]**

*Let $w^{\sigma }=(w_{1}^{\sigma },\;w_{2}^{\sigma },\;\ldots \;w_{n}^{\sigma })$ denote the weighting vector induced by the ordering $x_{\left(1\right)}\leq x_{\left(2\right)}\leq \cdots \leq x_{\left(n\right)}$, that is*,

$$w^{\sigma }=\left(\mu (H_{1})-\mu (H_{2}),\;\mu (H_{2})-\mu (H_{3}),\;\ldots ,\;\mu (H_{n})-0\right).$$

*Then, a capacity is said to be *buoyant* if for all orderings σ, it holds that $w_{i}^{\sigma }\leq w_{i+1}^{\sigma }$ for all i=1,…,n−1. A capacity is said to be *antibuoyant* if instead it holds that $w_{i}^{\sigma }\geq w_{i+1}^{\sigma }$ for all i=1,…,n−1*.

The recently proposed delay-averse capacities [17] are characterized similarly to antibuoyant capacities, but instead require decreasing weights based on the source, that is, the effective weight applied to $w_{1}$ is always greater than $w_{2}$ and so on. These are geared towards temporal data, so that more recent inputs can be allocated more importance while still accounting for variable interactions.

In the following section, we show how antibuoyancy can be used to help construct measures of species diversity.

## 4. Species diversity based on antibuoyant capacities

The concepts of diversity and conservation value may sometimes be conflated, with some measures in use clearly aiming at a trade-off between overall abundance and characteristics that seem appealing when it comes to conservation. A number of species diversity indices (including Simpson's index) can be interpreted as special instances of quasi-arithmetic means [1] or aggregation functions more generally.

Both the Sugeno and Choquet integral are examples of aggregation functions exhibiting average-like behaviour. The result of applying the functions to sets of inputs always results in outputs in the same range as the inputs, and increasing an input never results in decreases to the output.

A prominent example of less well-known aggregation functions applied to species diversity is the use of the geometric mean, which has been seen to act as a proxy for species diversity that is more sensitive to smaller values (corresponding with rare species) [18]. The aim is to still capture an idea of the number of individuals, but also to reward or inflate the value of those communities with species numbers more evenly distributed.

The geometric mean can be expressed as

$$GM(x_{1},x_{2},\ldots ,x_{n})={\left(\prod_{i=1}^{n}x_{i}\right)}^{1/n},$$

that is, it is the nth root of the product of all inputs.

When such a function is used to aggregate species abundances, we can see that redistributions from smaller to larger inputs will result in decreases to the output.

Suppose for a given set of inputs with $x_{1}<x_{2}$, we proportionally redistribute some of the value from $x_{1}$ to $x_{2}$.

$$\begin{array}{rcl} GM_{h}(x_{1}-h,x_{2}+h,\ldots ,x_{n}) & = & {\left((x_{1}-h)(x_{2}+h)\prod_{i=3}^{n}x_{i}\right)}^{1/n}, \end{array}$$

from which we can determine that $GM_{h}<GM$ (i.e. the output will decrease and adhere to the Pigou–Dalton principle) whenever $(x_{1}-h)(x_{2}+h)<x_{1}x_{2}$, which is true for all $0<x_{1}<x_{2}$ and h>0.

We can also introduce weights into the geometric mean, so that the relative importance of species is accounted for. In this case, we have

$$GM_{w}(x_{1},x_{2},\ldots ,x_{n})=\prod_{i=1}^{n}x_{i}^{w_{i}},$$

where $\sum_{i=1}^{n}w_{i}=1,w_{i}\geq 0$.

However, with a fairly simple example, we can show that satisfaction of the Pigou–Dalton principle can be lost for this weighted version.

***Example 1*.**

Consider a community with two common species and one rare species. Measures of diversity are desired such that the rare species is allocated additional importance. We consider first a weighted geometric mean with weights w=(0.5,0.25,0.25), where the 0.5 value is allocated to the rare species. We have

$$\begin{array}{r} GM_{w}(10,5,20)=10^{0.5}\times 5^{0.25}\times 20^{0.25}=10. \end{array}$$

However, if we look at another community with $x_{1}=10-1=9$ and $x_{2}=5+1=6$, where we have redistributed abundance from a more abundant to less abundant species, we then have

$$GM_{w}(9,6,20)=9^{0.5}\times 6^{0.25}\times 20^{0.25}=9.23,$$

that is, the Pigou–Dalton progressive transfers principle has been violated.

On the other hand, we can consider a Choquet integral with respect to an antibuoyant capacity.

***Example 2*.**

(Cont'd from example 1) For the same community, we can instead consider a Choquet integral with respect to the antibuoyant capacity represented in figure 5.

**Figure 5.**
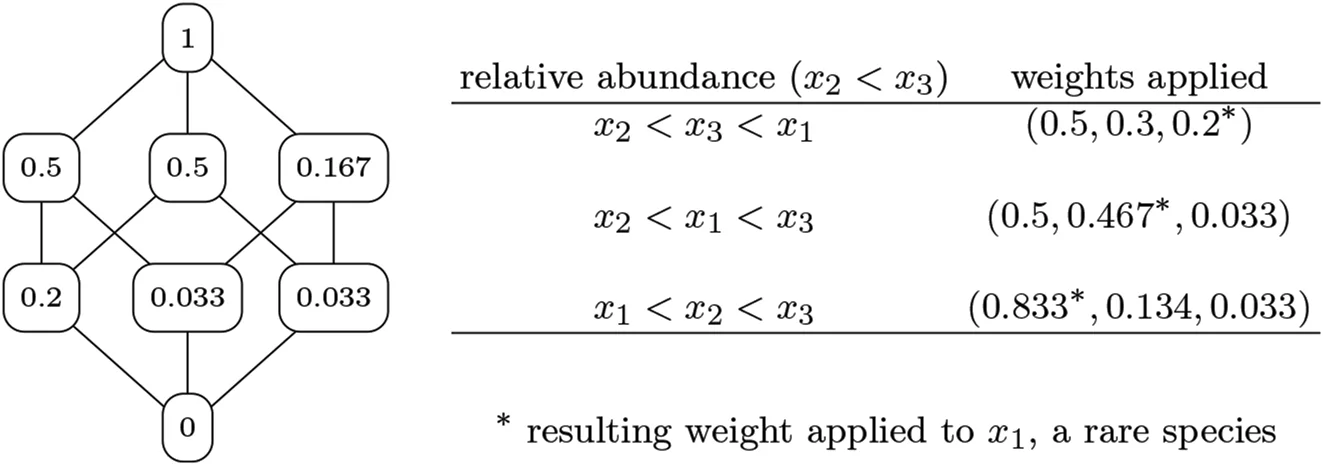
Antibuoyant capacity from example 2, where $x_{1}$ is the relative abundance of a rare species while $x_{2}$ and $x_{3}$ are considered equally important. More importance is placed on the rare species but the largest weights are always applied to the least abundant species.

The respective weighting vectors for when the rare species has the highest, second highest or lowest abundance are also shown, where ${}^{*}$ indicates the weight applied to the rare species.

In this case, we have $C_{\mu }(10,5,20)=0.5\times 5+0.467\times 10+0.033\times 20=7.83$ and $C_{\mu }(9,6,20)=0.5\times 6+0.467\times 9+0.033\times 20=7.863$, which satisfies the requirements of the Pigou–Dalton principle.

The Choquet integral has a number of useful properties in addition to being able to model in this behaviour. For example, scaling the species values by multiplying by a positive constant k results in the output being scaled by the same amount, and adding a positive value to each of the inputs also simply results in the output having the same amount added, that is, it is scale- and shift-invariant. This is not the case for the geometric mean.

As mentioned previously, the Choquet integral generalizes the OWA function. In the study of economic inequality and welfare indices, the OWA function with antibuoyant weights, that is, where the largest input is assigned the smallest weight, has been proposed as suitable for defining welfare indices [19], from which inequality indices can be constructed. Several of the classical indices, the Gini index, Bonferroni index and De Vergottini, were shown to be special cases in this framework.

There is less precedent for the application of the Sugeno integral; however, as an aggregation function, it has some interesting semantical interpretations that could make it appealing for some evaluations related to species diversity, which we will briefly discuss here.

From its calculation, we note that an output of y for the Sugeno integral indicates that there is a set of inputs $H_{i}$ such that both the capacity value $\mu (H_{i})$ and the inputs themselves $x_{\left(i\right)},x_{\left(i+1\right)}$ and so on, are all at least y. Along with the benefit of working on ordinal scales, the capacity values can hence be defined according to these semantics in the given context. Consider the following example.

***Example 3*.**

Suppose we consider an ordinal scale describing the health of a species within a community, using labels from the IUCN Red List Categories^1^: Least Concern, Near Threatened, Vulnerable, Endangered, Critically Endangered, Extinct in the Wild and Extinct.

At a community level, we can assign values to an ordinal capacity such that a community is only evaluated at the level of ‘Least Concern’ if all species are evaluated with this label, then is considered ‘Near Threatened’ if no more than 10% of the species are evaluated as ‘Vulnerable’, that is, at least 90% of species are either rated as ‘Least Concern’ or ‘Near Threatened’ and so on.

Unfortunately, the Sugeno integral does not exhibit behaviour consistent with the Pigou–Dalton principle. This becomes apparent when we observe that there may often be only one *active* input, with inputs above and below this not usually affecting the output. For example, in figure 4a, the active input is $x_{\left(2\right)}$. It is clear that redistributions from $x_{\left(1\right)}$ to $x_{\left(2\right)}$ will increase the input. Similarly in figure 4b, while transfers from $x_{\left(3\right)}$ to $x_{\left(2\right)}$ could indeed increase the input (once $x_{\left(2\right)}$ becomes greater than the current output $S_{\mu }$), transfers from $x_{\left(1\right)}$ to $x_{\left(2\right)}$ can achieve the same thing.

Nonetheless, the Sugeno integral (which can also be used to model indices like the h-index used for journals and researchers [20]) is a nice example of a measure that can take into account abundance of species as well as the importance of individuals and different groups of species. In ordinal settings (where the idea of redistributions of value may not be relevant), it provides a useful way of aggregating inputs that is readily interpretable, with an output of y indicating that there is a group of inputs, whose overall importance or significance is at least y, and whose ordinal values are all also at least the level of y.

## 5. Construction

We have already seen examples of antibuoyant capacities in the ecological setting. In this section, we provide some practical methods for constructing antibuoyant capacities more generally and for any number of species. As with previous sections, we may defer some of the details to appendix A, but hope to provide the key ideas behind the construction methods here so that such capacities can be more easily applied.

### 5.1. *p*-symmetric antibuoyant capacities

In [21], a method of generating antibuoyant capacities was presented that focuses on maximizing the value attributed to particular subsets, while still satisfying the necessary requirements. It turns out that such capacities belong to a simplified family known as p-symmetric capacities, which were introduced in [22]. While capacities usually require $2^{n}$ values to be specified when defining them, p-symmetric capacities treat members within specific subsets equally, and so only require $\left(|A_{1}|+1)(|A_{2}|+1)\cdots (|A_{p}|+1\right)$ values, where $A_{1},A_{2},\ldots ,A_{p}$ are distinct subsets (with cardinality $\left|A_{i}\right|$) called subsets of indifference, whose elements contained within are allocated equal importance. The antibuoyant capacities using the following approach have two subsets of indifference, A and N∖A. The steps below summarize algorithm 1 in [21].

1. Start with a table consisting of |A|+1 columns and |N∖A|+1 rows. The first row is prefilled with 0s and the entry in the last column of the last row is 1.
2. Allocate values to the last column so that the entries denote an increasing sequence with equal steps, that is, with $\frac{1}{\left|N\right|}$ added each time when going downwards.
3. Now starting with the second last column of the second row, moving downwards and then to the top of the previous column, allocate a value that is the average of the cell above and the cell to the right.
4. Once all entries have been filled, the resulting table provides the values for an antibuoyant capacity, with an entry in the ith row and jth column being the subset value for any set containing i−1 elements from N∖A and j−1 elements from A.

Example 4 is adapted from [21].

***Example 4*.**

Let us construct a capacity defined on {1,2,3,4,5} using the above approach where the set A consists of the elements {1,2,3}. Results of following the steps are shown in figure 6.

**Figure 6.**
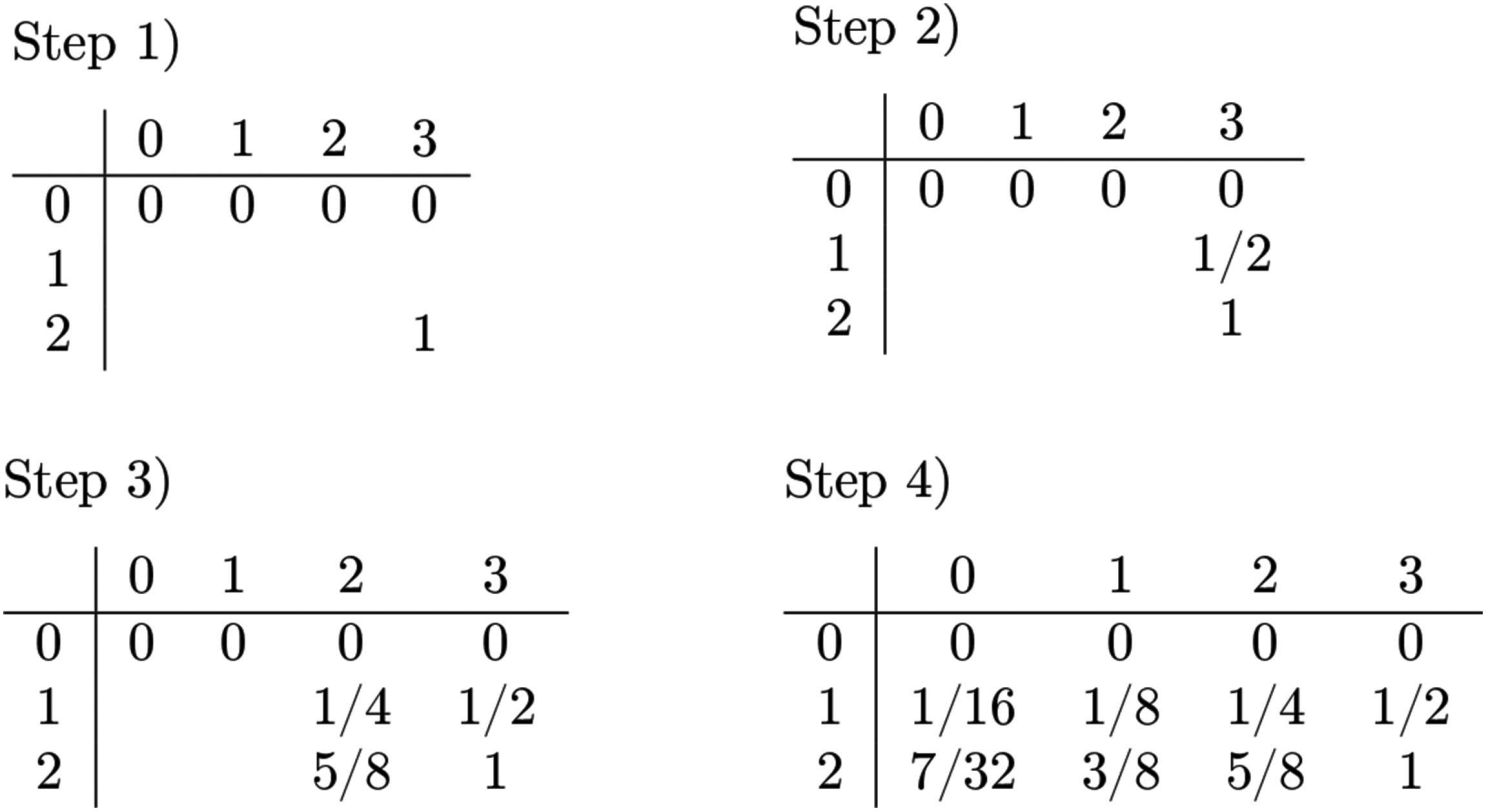
Construction of the antibuoyant p-symmetric capacity in example 4.

1. We have |A|=3 and N∖A=2, so we construct a (2+1)×(3+1) table and fill the first rows with zero and last cell with 1.
2. We then fill down the last column with equal steps.
3. Starting on the second row and the second last column (the cell corresponding with two elements from A and one element not from A), we assign the average of the cell to the right and above.
4. This continues until we complete the entire matrix.

After the matrix is obtained, the corresponding capacity values are determined based on how many of the elements come from A and how many come from N\A. For example, the set {1,2,4} has two elements from A and so would be the cell in the third column and the second row, hence μ({1,2,4})=1/4.

Weighting vectors corresponding with these capacities will have zero weights applied to the largest inputs as soon as there are only elements of A left. For example, for the ordering $x_{5}<x_{3}<x_{4}<x_{1}<x_{2}$, the effective weighting vector applied will be (1/2,1/4,1/4,0,0). In the context of species diversity, these capacities therefore favour the elements not included in A, that is, $x_{4}$ and $x_{5}$ here.

### 5.2. Random generation, linear combinations and optimization

The method in the previous section can be used to add or decrease the importance given to certain subgroups. If the differences are too extreme for the application, we can take weighted combinations of these methods with symmetric capacities or even with the additive and symmetric capacity, which allocates equal importance to all individuals. This is possible because the set of antibuoyant capacities is closed under weighted combinations, that is, if $\mu_{1},\mu_{2},\ldots ,\mu_{k}$ are antibuoyant capacities, then so is $\mu^{*}=w_{1}\mu_{1}+w_{2}\mu_{2}+\cdots +w_{k}\mu_{k}$, with $\sum_{i=1}^{k}w_{i}=1$ and all weights greater than zero.

If we want other options, random generation is possible. A number of approaches have been developed, from fairly simple approaches in [10] to methods based on linear extensions (see [23]) in [21] and sorting approaches in [24].

One can use any of these methods and then use linear combinations until the desired balance of importance and antibuoyancy is reached. To evaluate the overall importance of each of the inputs, a calculation known as the Shapley value can be used, which essentially takes a weighted average of all effective weights that are applied in weighting vectors depending on the given input order. The calculation of the Shapley index, as well as other indices used for interpreting interactions between elements can be found in appendix A.

Rather than using random combinations, the values of an antibuoyant capacity can also be learnt using optimization. We can either optimize towards a desired set of individual species importances (e.g. based on the Shapley value) subject to the antibuoyancy properties, or we can find the capacity values that best model a given dataset. A study conducted in [25] took this empirical approach to the construction of species diversity indicators, fitting aggregation functions to intuitive human judgements of species diversity drawn from a survey displaying synthetic communities and asking participants to rank them in terms of species diversity. Antibuoyancy was not applied in that study; however, the fitting approach is the same, except that we add linear constraints requiring that for any pair of inputs i,j,i≠j and all subsets A⊆N∖{i,j}, it always holds that

μ(A∪{i,j})−μ(A∪{i})≤μ(A∪{i})−μ(A).

The number of such constraints required does grow exponentially; however, a number of methods for reducing the complexity of the problem have been explored, for example, in [21,24].

### 5.3. Capacities that are antibuoyant on average

A new subclass of capacities was proposed in [26], which are far simpler to define complexity-wise, and which approximate the behaviour of antibuoyant capacities. That is, while it is not necessarily the case that all effective weighting vectors based on the input ordering will be decreasing, it will be the case ‘on average’. This is useful for larger input sets, with the antibuoyant on average capacities being determined from the Shapley values and interaction indices for pairs. In this way, different importances can be allocated to different species, and the Choquet integral output can still be used as a proxy for species diversity, although the Pigou–Dalton principle might be violated for some orderings.

## 6. Optimizing species diversity with both land and human population constraints

In this section, we consider an example application, where an antibuoyant Choquet integral may provide a new perspective. We then provide an example on a subset of real data. This problem has been considered in [8], which we consider now taking antibuoyancy into account. We consider allocation $a_{j}$ of various land types j=1,…,m, characterized by their human population density $p_{j}$ as well as population densities of various species $s_{ij},i=1,\ldots ,n$. We aim to look at the best combination of land types that maximizes species diversity, subject to accommodating the required population.

(6.1) $$\begin{array}{rcl} \text{Maximize} & & C_{\mu }(s_{1},s_{2},\ldots ,s_{n}),\;\text{wrt}\;a\\ \text{s.t.} & & \sum_{j=1}^{m}p_{j}a_{j}=P\\ & & \sum_{j=1}^{m}a_{j}=1,\\ & & a_{j}\geq 0,\;j=1,\ldots ,m\\ & & s_{i}=\sum_{j=1}^{m}s_{ij}a_{j},\;i=1,\ldots ,n \end{array}$$

P is the human population density constraint, $p_{j}$ is the human population density for land type j and $s_{i}=\sum_{j=1}^{m}s_{ij}a_{j}$ is the species abundance for the ith species given the chosen land allocation.

Although the land-use and population constraints are co-monotone and this has some nice simplifications when the Choquet integral is defined with respect to an antibuoyant capacity, in this case, the Choquet integral is acting on $s_{i}$, which are dependent on $a_{j}$, rather than aggregating $a_{j}$ as they are. In the case of general capacities, this is a difficult problem to solve, with one approach requiring one to solve an optimization problem for each ordering, that is

(6.2) $$\begin{array}{rcl} \text{Maximize} & & \sum_{i=1}^{n}w_{i}^{\sigma }s_{i}^{\sigma },\;\text{wrt}\;a,\sigma \\ \text{s.t.} & & \sum_{j=1}^{m}p_{j}a_{j}=P\\ & & \sum_{j=1}^{m}a_{j}=1,\\ & & a_{j}\geq 0,\;j=1,\ldots ,m\\ & & s_{i}=\sum_{j=1}^{m}s_{ij}a_{j},\;i=1,\ldots ,n\\ & & s_{i}^{\sigma }\leq s_{i+1}^{\sigma },\;i=1,\ldots ,n-1, \end{array}$$

where σ denotes a permutation of the inputs and $w^{\sigma }$ is the corresponding weighting vector applied in the Choquet integral depending on that permutation.

This is tractable for a moderate number of species, since the optimization problem itself is fairly simple, requiring only n+m non-negative decision variables and 2n+m+1 constraints. Depending on the data, there may also be further simplifications, for example, if some orderings are impossible, such as may be the case if some species are dominated by others in terms of abundance across all land types. Some may also be less likely, and so some heuristics may be able to reach useful solutions (see §6.2 following the example).

### 6.1. Example

We consider the bird survey dataset in [8] and the subset that was used in [7]. Reporting rates for a number of bird species were calculated based on field observations for 28 landscapes across the wider city of Melbourne in Australia. The land types are classified according to the human population density in terms of population per hectare based on official census data and the reporting rates were based on four visits to each site.

A subsample of four species is included in table 1, with the reporting rates at five population densities. The response curves for these bird species are shown together in figure 7, where the differences in how they interact with the human population become apparent.

**Figure 7.**
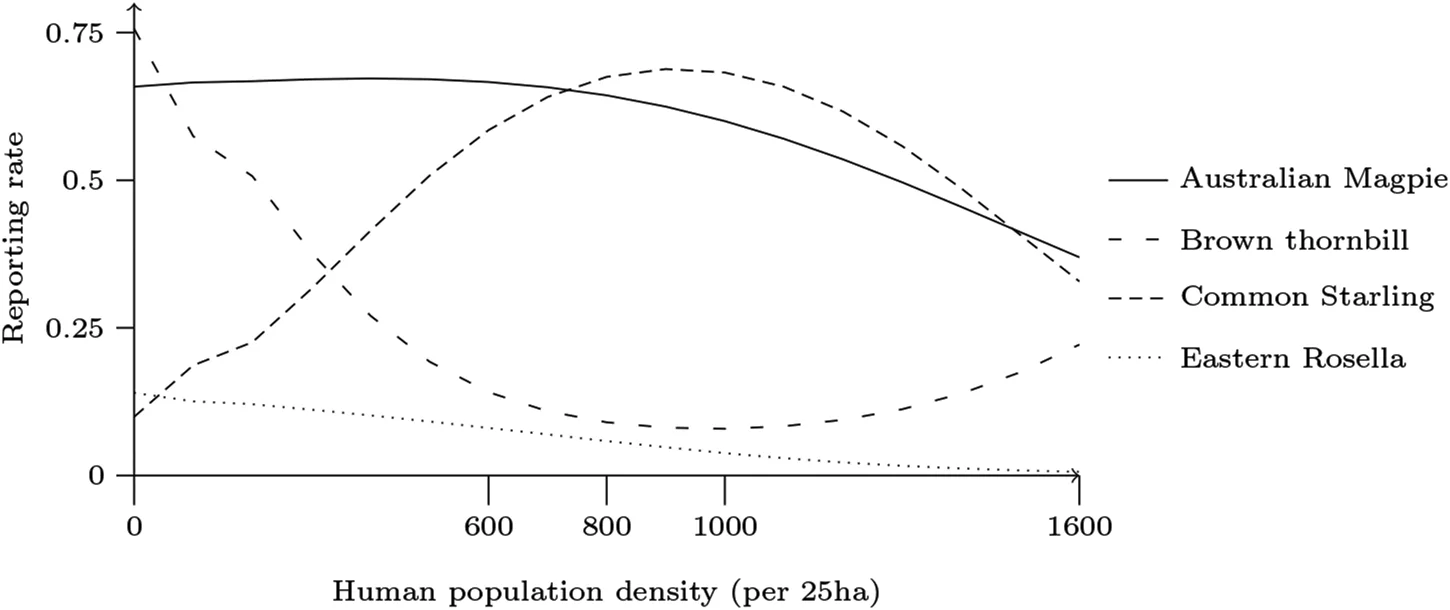
Response curves for the four species in table 1. The common starling gains an advantage from the presence of humans, whereas the other species are rarer for higher population densities.

**Table 1.** Reporting rates for different land types.

human population density	0	600	800	1000	1600
Australian magpie	0.658	0.666	0.644	0.600	0.370
brown thornbill	0.756	0.141	0.090	0.079	0.222
common starling	0.099	0.585	0.675	0.682	0.329
eastern rosella	0.140	0.081	0.058	0.038	0.006

The reporting rates can be interpreted as the probability of a species occurring at a given population density. For many birds, denser human populations make it more difficult for the species to thrive, while for others, being in the proximity of humans can actually be beneficial.

In our optimization problem, we consider how best to spread a human population of P=2.744 million across 3855 hectares (used to scale the sum of $a_{j}$) in a way that maximizes species diversity. We can maximize the use of high-density housing areas so that large conservation reserves are available, or we can spread out the population more evenly, with more medium density housing but fewer reserves. This is one aspect of the problem faced by governments and local councils in city planning.

In [7], three measures of species diversity were used as optimization objectives: raw abundance (i.e. the total population of birds regardless of species), the geometric mean of abundances and Shannon's entropy. Each of the measures resulted in slightly different optimal land-type allocations and expected species abundances.

We now apply the Choquet integral with respect to antibuoyant capacities to this problem with the same constraints. We use three different antibuoyant capacities, summarized in table 2. All are designed to provide additional weight or importance to the eastern rosella, which can be seen as a low occurring species across all land types.

**Table 2.** The three p-symmetric capacities used for optimization, including Shapley values.

antib. weights 1 ($v_{1}$), $\phi_{r}=0.46875$				
	no. of elements from A∖{r}			
{r}	0	1	2	3
0	0	0	0	0
1	0.125	0.25	0.5	1

antib. weights 2 ($0.8v_{1}$ + $0.2v_{2}$), $\phi_{r}=0.425$				
	no. of elements from A∖{r}			
{r}	0	1	2	3
0	0	0.040	0.084	0.14
1	0.140	0.284	0.54	1

antib. weights 3 ($0.6v_{1}$ + $0.4v_{2}$), $\phi_{r}=0.38125$				
	no. of elements from A∖{r}			
{r}	0	1	2	3
0	0	0.080	0.168	0.28
1	0.155	0.318	0.58	1

First, we construct a capacity using the method for defining p-symmetric antibuoyant capacities in §5.1. In this case, n=4 and our subset A corresponds with the subset consisting of the Australian magpie, brown thornbill and common starling. This capacity, which we will label AW1, assigns an overall importance of 0.45875 to the eastern rosella (according to the Shapley value calculation, $\phi_{r}$). We then construct two other antibuoyant capacities by taking linear combinations with a symmetric antibuoyant capacity, which (regardless of the relative abundance order) assigns weights of 0.4 to the species with the lowest population, 0.3 to the second lowest, 0.2 to the third lowest and 0.1 to the most abundant. The capacity AW2 takes a linear combination of 0.8 multiplied by the antibuoyant p-symmetric capacity and 0.2 multiplied by the symmetric capacity, while AW3 uses 0.6 and 0.4, respectively. The resulting importance allocated to the rare eastern rosella species with these capacities is 0.425 and 0.38125.

Note that these linear combinations are also p-symmetric and antibuoyant, with the capacity values for different subsets all read the same way from table 2, that is, we use row 1 if the rare species is included and row 2 otherwise, with the column indicating how many of the other species are included in the subset.

The results of optimization are summarized in tables 3 and 4. For each of the species diversity measures, table 3 provides the optimal land allocation proportions and the resulting expected species counts, rounded to whole numbers, with $s_{1},s_{2},s_{3},s_{4}$ corresponding, respectively, with the counts for the Australian magpie, brown thornbill, common starling and eastern rosella.

**Table 3.** Decision variable outputs following optimization.

	land types					species			
	0	600	800	1000	1600	$s_{1}$	$s_{2}$	$s_{3}$	$s_{4}$
abundance (RA)	0.110	0	0.890	0	0	2488	631	2358	260
geometric mean (GM)	0.358	0	0	0.525	0.117	2290	1304	1666	273
Shannon entropy (Sh)	0.350	0.328	0	0	0.322	2190	1474	1282	299
antib. weights 1 (AW1)	0.555	0	0	0	0.445	2043	1999	776	310
antib. weights 2 (AW2)	0.312	0.389	0	0	0.299	2217	1376	1376	296
antib. weights 3 (AW3)	0.403	0	0	0.405	0.192	2234	1463	1463	281

**Table 4.** Objective values depending on optimization method used.

	RA	GM	Sh	AW1	AW2	AW3
abundance (RA)	5736	990.0	1.1106	259.81	410.52	561.23
geometric mean (GM)	5534	1079.6	1.2156	272.87	430.84	588.82
Shannon entropy (Sh)	5244	1054.3	1.2289	298.65	446.21	586.40
antib. weights 1 (AW1)	5128	995.9	1.1894	310.33	440.47	570.62
antib. weights 2 (AW2)	5266	1056.3	1.2276	296.48	442.85	589.23
antib. weights 3 (AW3)	5441	1077.0	1.2250	281.43	438.63	595.83

All measures other than raw abundance allocate a high proportion of land to conservation reserves (i.e. spaces with no human population), with AW1 having the highest value here, since this capacity allocates very high importance to the eastern rosella. We can see that the other antibuoyant capacities, AW2 and AW3, yield similar results to Shannon's entropy and the geometric mean, respectively.

The objectives reached for each of the species diversity measures are shown in table 4, with the row labels indicating the optimization method used, and the columns indicating the calculations for each measure. The results here support the idea that all measures work towards similar ideals, but from these and the results in table 3, it is clear that varying the choice of species diversity calculation can have a noticeable impact on the outcomes.

### 6.2. Potential improvements to optimization efficiency

For AW1, AW2 and AW3, we considered all potential orderings in the optimization process. An approach to limiting the number of orderings that need to be checked is to look at the upper and lower limits of each species. We have, for each species

$$\begin{array}{rcl} \max or\min & & \sum_{j=1}^{n}w_{j}s_{ij}\\ \text{s.t.} & & \sum_{j=1}^{n}w_{j}=1\\ & & \sum_{j=1}^{n}w_{j}p_{j}\geq P, \end{array}$$

which is a linear programming problem. The solutions to the max and min versions will give the upper and lower possible values for $s_{i}$ respectively. Depending on how much these intervals overlap, only certain orders may need checking. For example, with the species considered here, the eastern rosella achieves a maximum population of 310 (obtained using AW1), which is far below the values for other species, so we could have considered only orderings that have $s_{4}$ as the lowest input to the Choquet integral. Some similar observations at the other end of the scale could be made regarding the Australian magpie, and so in general, we could have considered only the orderings with $s_{1}\geq s_{2}\geq s_{3}\geq s_{4}$ and $s_{1}\geq s_{3}\geq s_{2}\geq s_{4}$ rather than all 24.

### 6.3. Optimizing species diversity with simpler resource allocation constraints

A simpler problem that we briefly detail here is where we consider maximizing species diversity subject to a single resource allocation constraint, that is

(6.3) $$\begin{array}{rcl} \text{Maximize} & & \sum_{i=1}^{n}w_{i}^{\sigma }s_{i}^{\sigma },\;\text{wrt}\;a\\ \text{s.t.} & & \sum_{i=1}^{n}a_{i}s_{i}\leq R\\ & & a_{j}\geq 0,\;i=1,\ldots ,n \end{array}$$

The finite resource might be land, food or water required per individual, denoted $a_{i}$. We could even consider an alternative (and much simpler) version of (6.1), where we instead consider the sustainable human population density associated with each species. In this case, the constraint, $\sum_{i=1}^{n}a_{i}s_{i}$ would be the human population constraint. A key difference here is that we are directly including $s_{i}$ as decision variables in our objective function.

If problems can be formulated in this way, solutions are far easier to obtain, with it being established in [9] that the optimum allocation is found on the simplex corresponding with the relative order of the $a_{i}$ values. That is, if the data are indexed so that $a_{1}\leq a_{2}\leq \cdots \leq a_{n}$, then we can optimize only on the simplex $s_{1}\geq s_{2}\geq \cdots \geq s_{n}$. This means that the highest level of biodiversity is obtained when the species requiring the most resources is as large as possible.

## 7. Conclusion

In this paper, we have proposed the use of Choquet integrals with respect to antibuoyant capacities for providing a measure of species diversity that can allocate varying weight to different species but also satisfies the Pigou–Dalton principle of progressive transfers. We also considered potential applications of the Sugeno integral. We provided an overview of some important results and their implications for this application area, as well as providing an example application with some optimization results for real data relating to species diversity of birds and how this can be affected by human population density in the land-use allocation problem.

This problem brings some additional challenges to the optimization problems considered so far for antibuoyant capacities, since the effective inputs to the Choquet integral are not used directly as optimization decision variables, relying on auxiliary variables that denote the species populations for different land types. Considering such challenges and how to provide additional efficiency in this space is hence an opportunity for further research. There may also be useful applications in this domain for the related concept of ‘temporal antibuoyancy’ mentioned in §3, which could allow for changes in biodiversity over time to be modelled.

## Supplementary Material



## Data Availability

Optimization algorithms and data are included in the supplementary R code file. The data itself used are included in tables in the paper. Supplementary material is available online [27].

## Footnote

^1^

http://www.iucnredlist.org/

## Theme

One contribution of 9 to a theme issue ‘Data driven modelling for living systems’.

## Appendix A

The following definitions facilitate a deeper mathematical understanding of the concepts presented throughout the paper. We provide them here for those interested in these details.

### A.1. Representation of capacities

The Möbius representation of capacities can be useful for simplifying calculations and optimization solutions, since a number of simplifying assumptions can be introduced that result in sparse measures in this representation.

Definition 5. [28]

*For a set function μ, its Möbius representation is given for each set A⊆N by*

$$M^{\mu }(A)=\sum_{B\subseteq A}(-1{)}^{\left|A\setminus B\right|}\mu (B).$$

The inverse calculation, known as the Zeta transform, is given by

$$\mu (A)=\sum_{B\subseteq A}M^{\mu }(B).$$

In terms of the Möbius transform, the Choquet integral can be expressed as

(A 1) $$C_{\mu }(x)=\sum_{A\subseteq N}M^{\mu }(A)\min_{i\in A}x_{i}.$$

### A.2. Ordered weighted averaging and the buoyancy property

The Choquet integral with respect to a symmetric capacity is the OWA function [29].

Definition 6. [15]

*For a given input x, let $x_{\left(1\right)}\geq x_{\left(2\right)}\geq \cdots \geq x_{\left(n\right)}$ denote a non-increasing permutation of the inputs. The OWA operator is given by*

$$\mathrm{OWA}(x)=\sum_{i=1}^{n}w_{i}x_{\left(i\right)}.$$

*Definition 7*.
*A weighting vector $w,\sum_{i=1}^{n}w_{i}=1,w_{i}\geq 0$ associated with an OWA function is said to be buoyant if $w_{i}\geq w_{j}$ whenever i<j. It is said to be extensive [15] if $w_{i}>w_{j}$ whenever i<j*.

### A.3. Interaction and cardinality indices

The following indices are useful for interpreting capacities. The Shapley value gives the average importance and can be interpreted similarly to the weights of a mean, with the values adding up to 1. The interaction indices indicate whether groups of inputs result have a synergistic or redundancy effect.

*Definition 8*. [30]
*Let μ be a capacity on N. The Shapley index for every i∈N is*

$$\phi_{i}=\phi (i)=\sum_{A\subseteq N\setminus \{i\}}\frac{(n-|A|-1)!|A|!}{n!}[\mu (A\cup \{i\})-\mu (A)].$$

*The Shapley value is the vector ϕ(μ)=(ϕ(1),…,ϕ(n))*.

*Definition 9*.
*Let μ be a capacity. The pairwise interaction index for every pair i,j∈N is [31]*

$$I_{ij}=\sum_{A\subseteq N\setminus \{i,j\}}\frac{(n-|A|-2)!|A|!}{(n-1)!}[\mu (A\cup \{i,j\})-\mu (A\cup \{i\})-\mu (A\cup \{j\})+\mu (A)].$$

*Definition 10*. [32,33]
*Let μ be a capacity. The Shapley interaction index for every set A⊆N is*

$$I(A)=\sum_{B\subseteq N\setminus A}\frac{(n-|B|-|A|)!|B|!}{(n-|A|+1)!}\sum_{C\subseteq A}(-1{)}^{\left|A\setminus C\right|}\mu (B\cup C).$$

In Möbius representation, the Shapley value and interaction indices are written as [34]

$$\begin{array}{rl} \phi (i)= & \sum_{B|\;i\in B}\frac{1}{|B|}M(B),\\ I(A)=\sum_{B|A\subseteq B} & \frac{1}{|B|-|A|+1}M(B). \end{array}$$

### A.4. Buoyancy and antibuoyancy

Buoyancy can also be expressed for all A⊆N∖{i,j}, and for each pair i,j∉A,i≠j

(A 2) μ(A∪{i,j})−μ(A∪{j})≤μ(A∪{j})−μ(A).

In Möbius representation, the necessary and sufficient condition for buoyancy is [9]

(A 3) $$\sum_{\overset{B\subseteq A\cup \{i,j\}}{\{i,j\}\subseteq B}}M^{\mu }(B)\leq \sum_{\overset{B\subseteq A\cup \{i\}}{i\in B}}M^{\mu }(B)-\sum_{\overset{B\subseteq A\cup \{j\}}{j\in B}}M^{\mu }(B),$$

for all i,j∈N and all A⊆N∖{i,j}. In addition, the condition

$$\sum_{\overset{B\subseteq N}{i\in B}}M^{\mu }(B)\geq 0,\forall i\in N,$$

characterizes monotonicity, that is, it ensures that none of the $w_{i}^{\sigma }$ are negative.

In [24], it was shown that for Sugeno λ-fuzzy measures [13], which satisfy μ(A∪B)=μ(A)+μ(B)+λμ(A)μ(B), antibuoyancy will hold if and only if for all i,j∈N we have

(A 4) $$\frac{\mu (\{j\})}{1+\lambda \mu (\{j\}}\leq \mu (\{i\}).$$
